# On the Dotsenko–Fateev complex twin of the Selberg integral and its extensions

**DOI:** 10.1007/s11139-023-00804-3

**Published:** 2023-12-29

**Authors:** Yury A. Neretin

**Affiliations:** 1 Fakultät für Mathematik, Universität Wien, Wien, Austria; 2 Institute for Information Transmission Problems, Moscow, Russia; 3 Department of Mechanics and Mathematics, Moscow State University, Moscow, Russia

**Keywords:** Gamma function, Beta integrals, Selberg integrals, Hypergeometric functions, 33B15, 33C70

## Abstract

The Selberg integral has a twin (‘the Dotsenko–Fateev integral’) of the following form. We replace real variables $x_k$ in the integrand $\prod |x_k|^{\sigma -1}\,|1-x_k|^{\tau -1} \prod |x_k-x_l|^{2\theta }$ of the Selberg integral by complex variables $z_k$, integration over a cube we replace by an integration over the whole complex space ${\mathbb {C}}^n$. According to Dotsenko, Fateev, and Aomoto, such integral is a product of Gamma functions. We define and evaluate a family of beta integrals over spaces ${\mathbb {C}}^m\times {\mathbb {C}}^{m+1}\times \dots \times {\mathbb {C}}^n$, which for $m=n$ gives the complex twin of the Selberg integral (with three additional integer parameters).

## Results of the paper

Recall that the Selberg integral (Selberg, 1944) is given by

1.1 $$\begin{aligned} S_n(\sigma ,\tau ;\theta ):= & {} \int _0^1\dots \int _0^1\prod _{j=1}^n |x_k|^{\sigma -1}|1-x_k|^{\tau -1} \prod _{1\le k<l\le n}|x_k-x_l|^{2\theta }\prod _{j=1}^n dx_j\nonumber \\= & {} n!\prod _{k=1}^n\frac{\Gamma (\sigma +(k-1)\theta )\,\Gamma (\tau +(k-1)\theta ) \,\Gamma (k\theta )}{\Gamma (\sigma +\tau +(n+k-2)\theta )\,\Gamma (\theta )}, \end{aligned}$$

see a detailed discussion in Andrews, Askey, Roy [1], Chapter 8, for original Selberg’s proof, see Lique, Thibon [2]. Dotsenko and Fateev (1985, [3], formula (B.9)) published (without a proof) the following identity, which looks like a twin of the Selberg integral,

1.2 $$\begin{aligned} S_n^{\mathbb {C}}(\sigma ,\tau ;\theta ):= & {} \int _{{\mathbb {C}}^n} \prod _{k=1}^n |z_k|^{2\sigma -2}|1-z_k|^{2\tau -2} \prod _{1\le k<l\le n}|z_k-z_l|^{4\theta } \prod _k d\textrm{Re}\, z_k\,d\textrm{Im}\, z_k\nonumber \\= & {} \frac{1}{n!}\prod _{j=1}^n\frac{ \sin \pi (\sigma +(j-1)\gamma )\,\sin \pi (\tau +(j-1)\theta )\,\sin \pi (j\theta )}{\sin \pi (\sigma +\tau +(n+j-2)\theta )\,\sin \pi (\theta )}\nonumber \\{} & {} \cdot S(\sigma ,\tau ;\theta )^2. \end{aligned}$$

This integral was rediscovered by Aomoto [4] (who also presented a proof), see also Mimachi, Yoshida [5] and a review of integrals of the Selberg type integrals by Forrester, Warnaar [6].

The domain of convergence of this integral is narrow, see [6],

1.3 $$\begin{aligned}&\textrm{Re}\,\sigma>0,\quad \textrm{Re}\,\tau>0, \quad \textrm{Re}\, \theta >-\tfrac{1}{n}, \end{aligned}$$

1.4 $$\begin{aligned}&\textrm{Re}\,(\sigma + (n-1)\theta )>0, \quad \textrm{Re}\,(\tau + (n-1)\theta )>0; \end{aligned}$$

1.5 $$\begin{aligned}&\textrm{Re}\,(\sigma +\tau +(n-1)\theta )<1,\quad \textrm{Re}\,(\sigma +\tau +2(n-1)\theta )<1. \end{aligned}$$

### Some notation

For a complex variable *z* we denote

$$\begin{aligned} \,d\,{\overline{\overline{z}}} = d\textrm{Re}\, z\,\,d\textrm{Im} z=\tfrac{1}{2i}dz\, d\overline{z}. \end{aligned}$$

By

$$\begin{aligned} {\textbf{a}}=a\bigl |a' \end{aligned}$$

we denote a pair of complex numbers such that $a-a'$ is integer. Denote by $\Lambda$ the set of such pairs. It splits into a countable family of complex lines $a-a'=k$, where *k* ranges in ${\mathbb {Z}}$. Denote

$$\begin{aligned} {{\varvec{1}}}:=1\bigl |1,\qquad {{\varvec{2}}}:=2\bigl |2, \qquad \lfloor {\textbf{a}}\rfloor =\tfrac{1}{2} \textrm{Re}\,(a+a'). \end{aligned}$$

For a complex *z* denote

$$\begin{aligned} z^{\textbf{a}}=z^a\,\overline{z}^{a'}=|z|^{a+a'} e^{i(\arg z)(a-a')}. \end{aligned}$$

In particular,

$$\begin{aligned} z^{{\varvec{1}}}=|z|^2,\qquad (-1)^{\textbf{a}}=(-1)^{a-a'}. \end{aligned}$$

### Gamma function of the complex field

Following Gelfand, Graev, and Retakh [7], we define the Gamma function of the complex field by

1.6 $$\begin{aligned} \Gamma ^{\mathbb {C}}({\textbf{a}})= & {} \Gamma ^{\mathbb {C}}(a|a'):= \frac{1}{\pi }\int _{\mathbb {C}}z^{{\textbf{a}}-{{\varvec{1}}}} e^{2i\textrm{Re}\, z} \,d\,{\overline{\overline{z}}} \nonumber \\:= & {} i^{a-a'} \frac{\Gamma (a)}{\Gamma (1-a')}= i^{a'-a} \frac{\Gamma (a')}{\Gamma (1-a)}= \frac{i^{a'-a}}{\pi }\Gamma (a)\,\Gamma (a')\sin \pi a'. \end{aligned}$$

The integral conditionally converges if $0<\lfloor {\textbf{a}}\rfloor <1$, it admits a meromorphic continuation to each complex line $a-a'=k$.

The *beta function of the complex field* is defined by

1.7 $$\begin{aligned} \mathrm B^{\mathbb {C}}({\textbf{a}},{\textbf{b}}):=\frac{1}{\pi }\int _{\mathbb {C}}z^{{\textbf{a}}-{{\varvec{1}}}} (1-z)^{{\textbf{b}}-{{\varvec{1}}}}\,\,d\,{\overline{\overline{z}}} = \frac{\Gamma ^{\mathbb {C}}({\textbf{a}})\,\Gamma ^{\mathbb {C}}({\textbf{b}})}{\Gamma ^{\mathbb {C}}({\textbf{a}}+{\textbf{b}})}. \end{aligned}$$

The domain of (absolute) convergence is

$$\begin{aligned} \lfloor {\textbf{a}}\rfloor>0,\quad \lfloor {\textbf{b}}\rfloor >0,\quad \lfloor {\textbf{a}}+{\textbf{b}}\rfloor <1. \end{aligned}$$

### Results of the paper

Denote by $\mathcal {Z}$ a collection of point configurations

$$\begin{aligned} z_{11}\in {\mathbb {C}},\, (z_{21},z_{22})\in {\mathbb {C}}^2, \dots , (z_{n1},\dots ,\,z_{nn})\in {\mathbb {C}}^n. \end{aligned}$$

Denote by $\mathcal {C}_n$ the set of all possible $\mathcal {Z}$, so $\mathcal {C}_n\simeq {\mathbb {C}}^{n(n+1)/2}$. Denote

$$\begin{aligned} \,d\,{\overline{\overline{\mathcal {Z}}}} :=\prod _{j=1}^n \prod _{\alpha =1}^j \,d\,{\overline{\overline{z}}} _{j\alpha }. \end{aligned}$$

Let $${\varvec{\sigma }}_1$$, ..., $${\varvec{\sigma }}_n$$, $${\varvec{\tau }}_1$$, ..., $${\varvec{\tau }}_n$$, $${\varvec{\theta }}_{11}$$, $$\{{\varvec{\theta }}_{2j}\}_{j=1,2}$$, ..., $$\{{\varvec{\theta }}_{(n-1)j}\}_{j=1,2,\dots ,n-1}\in \Lambda $$.

#### Theorem 1

The following identity holds

1.8 $$\begin{aligned}{} & {} \int \limits _{\mathcal {C}_{\,n}} \prod _{j=1}^{n-1}\prod _{\alpha =1}^j z_{j\alpha }^{{\varvec{\sigma }}_j-{\varvec{\sigma }}_{j+1}-{\varvec{\theta }}_{j\alpha }} (1-z_{j\alpha })^{{\varvec{\tau }}_j-{\varvec{\tau }}_{j+1}-{\varvec{\theta }}_{j\alpha }} \prod _{p=1}^n z_{np}^{{\varvec{\sigma }}_n-{{\varvec{1}}}}(1-z_{np})^{{\varvec{\tau }}_n-{{\varvec{1}}}}\nonumber \\{} & {} \quad \times \prod _{j=1}^{n-1} \frac{\prod \limits _{1\le \alpha \le j;\, 1\le p\le j+1}(z_{j\alpha }-z_{(j+1)p})^{{\varvec{\theta }}_{j\alpha }-{{\varvec{1}}}}}{\prod \limits _{1\le \alpha ,\beta \le j;\,\alpha \ne \beta }(z_{j\alpha }-z_{j\beta })^{{\varvec{\theta }}_{j\alpha }-{{\varvec{1}}}}} \prod _{1\le p<q\le n}(z_{np}-z_{nq})^{{{\varvec{1}}}}\,\, \,d\,{\overline{\overline{\mathcal {Z}}}} \nonumber \\{} & {} \quad =(-1)^{\sum _{j=1}^{n-1}\sum _{\alpha =1}^j {\varvec{\theta }}_{j\alpha }}\pi ^{n(n+1)/2} \prod _{j=1}^{n} j! \nonumber \\{} & {} \quad \times \prod _{1\le \alpha \le j\le n-1} \Gamma ^{\mathbb {C}}({\varvec{\theta }}_{j\alpha }) \prod _{j=1}^n\frac{\Gamma ^{\mathbb {C}}({\varvec{\sigma }}_j)\,\Gamma ^{\mathbb {C}}({\varvec{\tau }}_j)}{\Gamma ^{\mathbb {C}}({\varvec{\sigma }}_j+{\varvec{\tau }}_j+\sum _{1\le \alpha \le j-1}{\varvec{\theta }}_{(j-1)\alpha })}. \end{aligned}$$

The domain of convergence of the integral is

$$\begin{aligned} \lfloor {\varvec{\sigma }}_j\rfloor>0,\quad \lfloor {\varvec{\tau }}_j\rfloor>0, \quad \lfloor {\varvec{\theta }}_{j\alpha }\rfloor >0, \quad \lfloor {\varvec{\sigma }}_j+{\varvec{\tau }}_j+\sum _{1\le \alpha \le j-1}{\varvec{\theta }}_{(j-1)\alpha }\rfloor < 1. \end{aligned}$$

This integral admits a further extension. Denote by $\mathcal {C}_n^m$ the space ${\mathbb {C}}^m\times {\mathbb {C}}^{m+1}\times \dots \times {\mathbb {C}}^n$ for $m\le n$. We denote points of this space by

$$\begin{aligned} \mathcal {Z}:=\bigl ((z_{m1}, \dots , z_{mm}), \dots , (z_{n1}, \dots , z_{nn})). \end{aligned}$$

We denote $\,d\,{\overline{\overline{\mathcal {Z}}}} :=\prod _{j=m}^n \prod _{\alpha =1}^j \,d\,{\overline{\overline{z}}} _{j\alpha }$. Consider the following collection of parameters $\in \Lambda$:

$$\begin{aligned}&\widehat{\varvec{\sigma }}_n^m=\{{\varvec{\sigma }}_m, \dots ,{\varvec{\sigma }}_n\}=:\{{\varvec{\sigma }}_j\}_{j=m,\dots , n}, \qquad \widehat{\varvec{\tau }}^m_n=\{{\varvec{\tau }}_j\}_{j=m,\dots , n},\\&\widehat{\varvec{\theta }}_n^m=\{{\varvec{\theta }}_{j\alpha }\}_{j=m,\dots ,n-1;\, \alpha =1,\dots ,j},\qquad {\varvec{\nu }}. \end{aligned}$$

#### Theorem 2

Denote

1.9 $$\begin{aligned} J_n^m(\widehat{\varvec{\sigma }}_n^m,\widehat{\varvec{\tau }}_n^m;{\varvec{\nu }},\widehat{\varvec{\theta }}_n^m)= & {} \int _{\mathcal {C}_{\,n}^{\,m}}\prod _{j=m}^{n-1}\prod _{\alpha =1}^j z_{j\alpha }^{{\varvec{\sigma }}_j-{\varvec{\sigma }}_{j+1}-{\varvec{\theta }}_{j\alpha }}(1-z_{j\alpha }) ^{{\varvec{\tau }}_j-{\varvec{\tau }}_{j+1}-{\varvec{\theta }}_{j\alpha }}\nonumber \\{} & {} \cdot \prod _{p=1}^n z_{np}^ {{\varvec{\sigma }}_n-{{\varvec{1}}}}(1-z_{np})^{{\varvec{\tau }}_n-{{\varvec{1}}}} \times \nonumber \\{} & {} \times \prod _{1\le \alpha ,\beta \le m;\, \alpha \ne \beta } (z_{m\beta }-z_{m\alpha })^{{\varvec{\nu }}-\frac{{{\varvec{1}}}}{{{\varvec{2}}}}} \times \nonumber \\{} & {} \times \prod \limits _{j=m}^{n-1} \frac{ \prod \limits _{1\le \alpha \le j;\, 1\le p\le j+1} (z_{j\alpha }-z_{(j+1)p})^{{\varvec{\theta }}_{j\alpha }-{{\varvec{1}}}} }{ \prod \limits _{1\le \alpha ,\beta \le j;\,\alpha \ne \beta }(z_{j\alpha } -z_{j\beta })^{{\varvec{\theta }}_{j\alpha }-{{\varvec{1}}}} }\nonumber \\{} & {} \cdot \prod _{1\le p<q\le n} (z_{np}-z_{nq})^{{\varvec{1}}}\,\,d\,{\overline{\overline{\mathcal {Z}}}} . \end{aligned}$$

The following identity holds:

1.10 $$\begin{aligned}{} & {} J_n^m(\widehat{\varvec{\sigma }}_n^m,\widehat{\varvec{\tau }}_n^m;{\varvec{\nu }},\widehat{\varvec{\theta }}_n^m) = (-1)^{{\varvec{\nu }}m(m-1)/2+ \sum \limits _{j=m}^{n-1}\sum \limits _{\alpha =1}^j {\varvec{\theta }}_{j\alpha }}\,\, \pi ^{n(n+1)/2-m(m-1)/2} \prod \limits _{j=m}^n j!\,\nonumber \\{} & {} \quad \times \prod _{j=1}^{m-1} \frac{\Gamma ^{\mathbb {C}}({\varvec{\sigma }}_m+(m-j){\varvec{\nu }})\, \Gamma ^{\mathbb {C}}({\varvec{\tau }}_m+(m-j){\varvec{\nu }})\,\Gamma ^{\mathbb {C}}((j+1){\varvec{\nu }})}{\Gamma ^{\mathbb {C}}({\varvec{\sigma }}_m+{\varvec{\tau }}_m+(2m-j-1){\varvec{\nu }}) \,\Gamma ^{\mathbb {C}}({\varvec{\nu }}) }\nonumber \\{} & {} \quad \times \prod _{j=m}^{n-1} \prod _{\alpha =1}^j \Gamma ^{\mathbb {C}}({\varvec{\theta }}_{j\alpha }) \prod \limits _{j=m}^n\frac{\Gamma ^{\mathbb {C}}({\varvec{\sigma }}_{j})\, \Gamma ^{\mathbb {C}}({\varvec{\tau }}_{j})}{\Gamma ^{\mathbb {C}}({\varvec{\sigma }}_j+{\varvec{\tau }}_j +\sum _{\alpha =1}^{j-1}{\varvec{\theta }}_{(j-1)\alpha })}. \end{aligned}$$

The following conditions are sufficient for absolute convergence of the integral

1.11 $$\begin{aligned}&\lfloor {\varvec{\sigma }}_j\rfloor>0,\, \lfloor {\varvec{\tau }}_j\rfloor>0, \, \lfloor {\varvec{\theta }}_{j\alpha }\rfloor>0, \, \lfloor {\varvec{\sigma }}_j+{\varvec{\tau }}_j+\sum _{1\le \alpha \le j-1}{\varvec{\theta }}_{(j-1)\alpha }\rfloor < 1\,\,\text {for} j> m;\end{aligned}$$

1.12 $$\begin{aligned}&\lfloor {\varvec{\nu }}\rfloor >0, \,\, \lfloor m{\varvec{\nu }}\rfloor<1,\,\, \lfloor {\varvec{\sigma }}_m+{\varvec{\tau }}_m+2(m-1){\varvec{\nu }}\rfloor <1, \end{aligned}$$

the conditions (1.11) also are necessary.

#### Remark

The factor in the second line of the right-hand side of (1.9) has a similar factor in the denominator of the third line (for $j=m$), we can unite them, but the formula is more readable without this combination. $\boxtimes$

Next, we set $m=n$ in (1.9). Then two factors $\prod _{j=m}^{n-1}$ of the integrand disappear. We denote ${\varvec{\sigma }}:={\varvec{\sigma }}_n$, ${\varvec{\tau }}:={\varvec{\tau }}_n$, ${\varvec{\theta }}:={\varvec{\nu }}$, $z_j=z_{nj}$ (other parameters in our case are absent) and come to the following extended Dotsenko–Fateev–Aomoto integral:

#### Corollary 1

1.13 $$\begin{aligned}{} & {} \int \limits _{{\mathbb {C}}^n}\prod _{j=1}^n z_j^{{\varvec{\sigma }}-{{\varvec{1}}}} (1-z_j)^{{\varvec{\tau }}-{{\varvec{1}}}} \prod _{1\le i<j\le n}(z_i-z_j)^{2{\varvec{\theta }}}\prod _{j=1}^n \,d\,{\overline{\overline{z}}} _j\nonumber \\{} & {} \quad = (-1)^{{\varvec{\theta }}\cdot n(n-1)/2} n!\,\pi ^n\,\prod _{j=1}^n\frac{\Gamma ^{\mathbb {C}}({\varvec{\sigma }}+(j-1){\varvec{\theta }}) \,\Gamma ^{\mathbb {C}}({\varvec{\tau }}+(j-1){\varvec{\theta }})\,\Gamma ^{\mathbb {C}}(j{\varvec{\theta }})}{\Gamma ^{\mathbb {C}}({\varvec{\sigma }}+{\varvec{\tau }}+(n+j-2){\varvec{\theta }})\,\Gamma ^{\mathbb {C}}({\varvec{\theta }})}. \nonumber \\ \end{aligned}$$

Formally, this formula modulo, a constant factor can be obtained from the Selberg integral (1.1) by a substitution

1.14 $$\begin{aligned} x\mapsto z,\quad \sigma \mapsto {\varvec{\sigma }},\quad \tau \mapsto {\varvec{\tau }},\quad \theta \mapsto {\varvec{\theta }},\quad 1\rightarrow {{\varvec{1}}},\quad \Gamma \mapsto \Gamma ^{\mathbb {C}}. \end{aligned}$$

We also get 3 additional integer parameters in comparison to (1.2), namely, $\sigma -\sigma '$, $\tau -\tau '$, $\theta -\theta '$.

#### Remark

a) Our way of derivation is valid if we add the condition $\lfloor {\varvec{\theta }}\rfloor >0$ to (1.3)–(1.5), it is easy to omit it if we know the final formula.

b) The paper by Dotsenko and Fateev [3] contains another integral (B.10) over ${\mathbb {C}}^m\times {\mathbb {C}}^n$ which looks similar to the Selberg integral and our integrals. For this reason, it is natural to think that the story with beta integrals of this type is not finished. $\boxtimes$

### Complex beta integrals and complex hypergeometric functions

Notice that the integral (1.7) for $\mathrm B^{\mathbb {C}}({\textbf{a}},{\textbf{b}})$ is a twin of the classical Euler beta integral, this formula appeared in the book by Gelfand, Graev, Vilenkin [8], Section II.3.7, 1962, as an exercise (a careful proof is not completely obvious). Our formulas (1.8), (1.9)-(1.10) also are twins of formulas from Neretin [9]. It is well known that the Selberg integral interpolates matrix beta integrals^1^ of Hua Loo Keng type [12] with respect to the dimension $2\theta$ of a basic field (${\mathbb {R}}$, ${\mathbb {C}}$, or quaternions ${\mathbb {H}}$) and sizes of matrices. One of purposes of [9] was an interpolation of matrix beta integrals of more general types as Gindikin [13] and the author [14]. Another purpose was an interpolation of joint distributions of eigenvalues of growing Hermitian matrices (for stochastic processes generated by integrals from [9], see Cuenca [15], Assiotis, Najnudel [16]). Our calculations in Section 2 are twins of calculations in [9].

Now there are known many complex twins of real beta integrals, see Bazhanov, Mangazeev, Sergeev, [17], Kels [18, 19], Derkachov, Manashov [20, 21], Derkachov, Manashov, Valinevich [20, 22], Neretin [23], Sarkissian, Spiridonov [24]. Usually, these extensions are not automatic, often usual ways are non-available or meet serious analytic difficulties. For instance, if we know the Selberg integral (1.1), then we can evaluate the integrals

$$\begin{aligned} \int \limits _{{\mathbb {R}}^n} \prod _{k=1}^n x_k^{\sigma -1} e^{-ax_k}\! \prod _{1\le k<l\le n} \! |x_k-x_l|^{2\theta }\, dx \,\, \,\text {and}\,\, \int \limits _{{\mathbb {R}}^n} e^{-a\sum x_k^2} \prod _{1\le k<l\le n} |x_k-x_l|^{2\theta }\, dx \end{aligned}$$

by examination of asymptotics of (1.1) as $\textrm{Re}\, \tau \rightarrow \infty$ and $\textrm{Re}\,\sigma , \textrm{Re}\,\tau \rightarrow \infty$, respectively, see Andrews, Askey, Roy [1], Corollaries 8.2.2, 8.2.3. But we cannot do the same procedure with their complex twins

$$\begin{aligned} \int \limits _{{\mathbb {C}}^n} \prod _{k=1}^n z_j^{{\varvec{\sigma }}-{{\varvec{1}}}} e^{i\textrm{Re}\, z_j} \prod _{1\le k<l\le n} |z_k-z_l|^{2{\varvec{\theta }}}\, \,d\,{\overline{\overline{z}}} \,\, \text {and}\,\, \int \limits _{{\mathbb {C}}^n} e^{-i\textrm{Re}\,\sum z_k^2} \prod _{1\le k<l\le n} |z_k-z_l|^{2{\varvec{\theta }}}\, \,d\,{\overline{\overline{z}}} \end{aligned}$$

due to a narrow domain of convergence (1.3)–(1.5). Also these integrals are not absolutely convergent, our way of evaluation of the Dotsenko–Fateev integral formally can be applied to these integrals, but this leads to too dangerous manipulations with non-absolutely convergent integrals (cf. [9], Subsect. 2.7, 2.8).

On the other hand, the Selberg integral has different real forms, see [1], Chapter 8, Exercise 14, and [6], formula (2.6). Apparently, for the complex case we have only one Dotsenko–Fateev integral.

Another part of this story is hypergeometric functions of the complex field, which are a kind of a lost chapter of theory of hypergeometric functions,^2^ see Gelfand, Graev, Retakh [7], Ismagilov [25], Mimachi [26], Molchanov, Neretin [27], Derkachov, Spiridonov, [28], Neretin [29], Sarkissian, Spiridonov [30]. For instance, the counterpart of the Gauss hypergeometric function $_2F_1$ is the following integral:

$$\begin{aligned} {}_2F_1^{\mathbb {C}}\left[ \begin{matrix}{\textbf{a}},{\textbf{b}};{\textbf{c}}\end{matrix};z \right] := \frac{1}{\pi \mathrm B^{\mathbb {C}}({\textbf{a}},{\textbf{b}})}\int _{\mathbb {C}}t^{{\textbf{b}}-{{\varvec{1}}}}(1-t)^{{\textbf{c}}-{\textbf{b}}-{{\varvec{1}}}}(1-zt)^{-{\textbf{a}}}\,\,d\,{\overline{\overline{t}}} , \end{aligned}$$

defined in Gelfand, Graev, Retakh [7] (this integral is a precise twin of the Euler integral representation of the Gauss hypergeometric function $_2F_1$, see, e.g., [1], Sect.2.2). It admits an explicit evaluation as a quadratic expression with two summands with products of Gaussian hypergeometric functions, see [27], Theorem 3.9. Twins of generalized hypergeometric functions $_pF_q$ were defined in [29]. Apparently, the most of identities for usual hypergeometric functions $_pF_q$ have complex twins, as it is explained in [29].

It is natural to ask about complex twins of the Heckman–Opdam multivariate hypergeometric functions [31]. This note gives a reason for this question, at least for functions related to the root systems of type $A_n$ (i.e., to the Jack functions). Consider the space ${\mathbb {R}}^1\times {\mathbb {R}}^2\times \dots \times {\mathbb {R}}^n$ with coordinates $\mathcal {X}=\{x_{j\alpha }\}$, where $1\le \alpha \le j\le n$. Consider the polyhedral cone $\mathcal {R}_n\subset \prod _{j=1}^n {\mathbb {R}}^j$ consisting of $\mathcal {X}$ satisfying the interlacing conditions

$$\begin{aligned} x_{(j+1)1}\ge x_{j1} \ge x_{(j+1)2}\ge x_{j2}\ge x_{(j+1)3}\ge \dots \ge x_{(j+1)(j+1)}, \quad x_{nn}>0, \end{aligned}$$

see [9], Subsect 1.1. We fix $t_n\ge \dots \ge t_1>0$ and denote by $\mathcal {R}_n(t)$ the set of $\mathcal {X}\in \mathcal {R}_n$ satisfying the conditions $x_{n\alpha }=t_\alpha$ (i.e., we consider a section of the cone by an affine subspace). So we get a convex polyhedron in ${\mathbb {R}}^1\times \dots \times {\mathbb {R}}^{n-1}$.

According to Okounkov and Olshanski [32] (see also Kazarnovski-Krol [33]), the Jack functions admit integral representations of the form

1.15 $$\begin{aligned}{} & {} J_{\sigma _1,\dots ,\sigma _n}^\theta (t_1,\dots ,t_n)= C(\sigma ,\theta ) \prod _{\alpha =1}^n t_\alpha ^{\sigma _n+(n-1)\theta /2}\prod _{1\le \alpha<\beta \le n} (t_\beta -t_\alpha )^{1-2\theta } \nonumber \\{} & {} \quad \times \int _{\mathcal {R}_{\,n}(t)} \prod _{j=1}^{n-1}\prod _{\alpha =1}^j x_{j\alpha }^{\sigma _j-\sigma _{j+1}-\theta } \nonumber \\{} & {} \quad \times \prod _{j=1}^{n-1} \frac{\prod \limits _{1\le \alpha \le j;\,1\le p\le n} |x_{j\alpha }-x_{(j+1)p}|^{\theta -1}\Bigr |_{x_{n1}=t_1, \dots , x_{nn}=t_n}}{\prod \limits _{1\le \alpha <\beta \le j}|x_{j\alpha }-x_{j\beta }|^{2\theta -1}} \prod _{1\le \alpha \le j\le n-1}dx_{j\alpha }, \nonumber \\ \end{aligned}$$

where $C(\sigma ,\theta )$ is a normalization constant (a product of Gamma functions). For $\theta =1/2$, 1, 2 this formula gives spherical functions on the Riemannian symmetric spaces

1.16 $$\begin{aligned} {\text {GL}}(n,{\mathbb {R}})/\textrm{O}(n),\,\, {\text {GL}}(n,{\mathbb {C}})/\textrm{U}(n),\,\, {\text {GL}}(n,{{\mathbb {H}}})/\mathop {\textrm{Sp}}\nolimits (n), \end{aligned}$$

where ${\mathbb {H}}$ is the algebra of quaternions and $\mathop {\textrm{Sp}}\nolimits (n)$ is the quaternionic unitary group.^3^ For the space ${\text {GL}}(n,{\mathbb {C}})/{\mathrm U}(n)$, Gelfand and Naimark [34], Sect. 9, obtained an elementary expression, this work was an initial point for further calculations of such type.

It is natural to hope that Jack functions of the complex field can be obtained from this expression by the same substitution (1.14). It is natural to hope that they are eigenfunctions of two commuting families of the Sekiguchi operators^4^

$$\begin{aligned} D(u;{\varvec{\theta }})&=\prod _{1\le k<l\le n}(t_k-t_l)^{-1} \det \Bigl \{t_k^{n-l}\bigl (t_k\frac{\partial }{\partial t_k}+(n-l)\theta +u\bigr )\Bigr \}_{1\le k, l\le n};\\ \overline{D}(u';{\varvec{\theta }})&=\prod _{1\le k<l\le n}(\overline{t}_k-\overline{t}_l)^{-1} \det \Bigl \{\overline{t}_k^{n-l}\bigl (\overline{t}_k\frac{\partial }{\partial \overline{t}_k}+(n-l)\theta '+u'\bigr )\Bigr \}_{1\le k, l\le n} \end{aligned}$$

(cf., [27], Propositions 3.9, 3.11, 3.12, [29], Corollary 1.4). Other natural questions: does exist a complex twin of the Harish-Chandra hypergeometric transform? Are spherical distributions on the symmetric spaces^5^

$$\begin{aligned} {\text {GL}}(n,{\mathbb {C}})/\textrm{SO}(n,{\mathbb {C}}),\, {\text {GL}}(n,{\mathbb {C}})\times {\text {GL}}(n,{\mathbb {C}})/\textrm{diag} {\text {GL}}(n,{\mathbb {C}}),\, {\text {GL}}(2n, {\mathbb {C}})/\mathop {\textrm{Sp}}\nolimits (2n,{\mathbb {C}}) \end{aligned}$$

Jack functions of the complex field?.

## Evaluation of integrals

### The Dirichlet integral

The complex twin of the Dirichlet integral (see, e.g., [1], Theorem I.8.6)

$$\begin{aligned} \int \limits _{\begin{array}{c} t_1>0,\dots ,t_n>0\\ t_1+\dots +t_n<1 \end{array}} \prod \limits _{j=1}^{n} t_j^{a_j-1}\cdot \left( 1-\sum \limits _{j=1}^n t_j\right) ^{a_{n+1}-1} \,dt_1\dots dt_n =\frac{\prod \limits _{j=1}^{n+1}\Gamma (a_j)}{\Gamma (\sum \limits _{j=1}^{n+1} a_j\bigr )} \end{aligned}$$

is given by the following statement.

#### Proposition 1

Denote

2.1 $$\begin{aligned} D_n^{\mathbb {C}}({\textbf{a}}_1,\dots , {\textbf{a}}_{n+1}):=\int _{{\mathbb {C}}^n} \prod _{j=1}^{n} t_j^{{\textbf{a}}_j-{{\varvec{1}}}}\cdot \left( 1-\sum _{j=1}^n t_j\right) ^{{\textbf{a}}_{n+1}-{{\varvec{1}}}} \,d\,{\overline{\overline{t}}} _1\dots \,d\,{\overline{\overline{t}}} _n. \end{aligned}$$

Then

2.2 $$\begin{aligned} D_n^{\mathbb {C}}({\textbf{a}}_1,\dots , {\textbf{a}}_{n+1})= \pi ^n \frac{\prod \limits _{j=1}^{n+1}\Gamma ^{\mathbb {C}}({\textbf{a}}_j)}{\Gamma ^{\mathbb {C}}\left( \sum \limits _{j=1}^{n+1} {\textbf{a}}_j\right) }, \end{aligned}$$

the conditions of convergence are

$$\begin{aligned} \lfloor {\textbf{a}}_j\rfloor >0, \qquad \left\lfloor \sum {\textbf{a}}_j\right\rfloor <1. \end{aligned}$$

#### Proof

Notice that for $u\in {\mathbb {C}}$ we have

2.3 $$\begin{aligned} \frac{1}{\pi }\int _{\mathbb {C}}t^{{\textbf{a}}-{{\varvec{1}}}} (u-t)^{{\textbf{b}}-{{\varvec{1}}}}\,\,d\,{\overline{\overline{t}}} _1\dots \,d\,{\overline{\overline{t}}} _n= u^{{\textbf{a}}+{\textbf{b}}-{{\varvec{1}}}}\, \frac{\Gamma ^{\mathbb {C}}({\textbf{a}})\,\Gamma ^{\mathbb {C}}({\textbf{b}})}{\Gamma ^{\mathbb {C}}({\textbf{a}}+{\textbf{b}})}. \end{aligned}$$

Indeed, the substitution $t=uz$ reduces this integral to (1.7).

Next, we integrate with respect to $t_n$ in (2.1), for this purpose in (2.3) we set $t=t_n$, $u=1-t_1-\dots -t_{n-1}$. We come to

$$\begin{aligned} D_n^{\mathbb {C}}({\textbf{a}}_1,\dots , {\textbf{a}}_{n+1})= \pi \, \frac{\Gamma ^{\mathbb {C}}({\textbf{a}}_n)\,\Gamma ^{\mathbb {C}}({\textbf{a}}_{n+1}) }{\Gamma ^{\mathbb {C}}({\textbf{a}}_n+{\textbf{a}}_{n+1})}\cdot D_{n-1}^{\mathbb {C}}({\textbf{a}}_1,\dots ,{\textbf{a}}_{n-1},{\textbf{a}}_n+ {\textbf{a}}_{n+1}). \end{aligned}$$

Iterating this identity, we get (2.2). $\square$

### The main lemma

#### Lemma 1

2.4 $$\begin{aligned}{} & {} \int _{{\mathbb {C}}^n} \prod _{p=1}^n u_p^{{\varvec{\sigma }}-{{\varvec{1}}}} (1-u_p)^{{\varvec{\tau }}-{{\varvec{1}}}} \prod _{1\le \alpha \le n-1;\, 1\le p\le n} (u_p-z_\alpha )^{{\varvec{\theta }}_\alpha -{{\varvec{1}}}} \prod _{1\le p<q\le n}(u_p-u_q)^{{{\varvec{1}}}}\,\,d\,{\overline{\overline{u}}} \nonumber \\{} & {} \quad =(-1)^{\sum \limits _{\alpha =1}^{n-1} {\varvec{\theta }}_\alpha }\,\pi ^n n! \frac{\Gamma ^{\mathbb {C}}({\varvec{\sigma }})\,\Gamma ^{\mathbb {C}}({\varvec{\tau }})\prod \limits _{\alpha =1}^{n-1} \Gamma ^{\mathbb {C}}({\varvec{\theta }}_\alpha )}{\Gamma ^{\mathbb {C}}\left( {\varvec{\sigma }}+{\varvec{\tau }}+\sum \limits _{\alpha =1}^{n-1}{\varvec{\theta }}_\alpha \right) } \nonumber \\{} & {} \quad \times \prod _{\alpha =1}^{n-1} z_\alpha ^{{\varvec{\sigma }}+{\varvec{\theta }}_\alpha -{{\varvec{1}}}} (1-z_\alpha )^{{\varvec{\tau }}+{\varvec{\theta }}_\alpha -{{\varvec{1}}}} \prod _{1\le \alpha ,\beta \le n-1;\, \alpha \ne \beta }(z_\beta -z_\alpha )^{{\varvec{\theta }}_\alpha -\frac{{{\varvec{1}}}}{{{\varvec{2}}}}}. \end{aligned}$$

The domain of convergence is

$$\begin{aligned} \lfloor {\varvec{\sigma }}\rfloor>0,\quad \lfloor {\varvec{\tau }}\rfloor>0, \quad \lfloor {\varvec{\theta }}_\alpha \rfloor >0, \quad \lfloor {\varvec{\sigma }}+{\varvec{\tau }}+\sum _{\alpha =1}^{n-1}{\varvec{\theta }}_\alpha \rfloor <1. \end{aligned}$$

#### Remark

The last factor in the right-hand side of (2.4) can be represented in the form

$$\begin{aligned} \prod _{1\le \alpha ,\beta \le n-1;\, \alpha \ne \beta } \!\!(z_\beta -z_\alpha )^{{\varvec{\theta }}_\alpha } \prod _{1\le \alpha<\beta \le n-1}\!\!(z_\beta -z_\alpha )^{-{{\varvec{1}}}} = \pm \prod _{1\le \alpha <\beta \le n-1}\!\!(z_\beta -z_\alpha )^{{\varvec{\theta }}_\alpha +{\varvec{\theta }}_{\beta }-{{\varvec{1}}}}. \end{aligned}$$

#### Proof

Step 1. We define new variables $x_1$, ..., $x_{n-1}$, $y\in {\mathbb {C}}$ instead of $u_p$ by

2.5 $$\begin{aligned} x_\alpha&=-\frac{\prod \limits _{1\le p\le n}(u_p-z_\alpha )}{\prod \limits _{1\le \beta \le n-1, \beta \ne \alpha }(z_\beta -z_\alpha )}; \end{aligned}$$

2.6 $$\begin{aligned} y:&=\sum _{1\le p\le n} u_p- \sum _{1\le \beta \le n-1} z_\beta , \end{aligned}$$

this transformation is similar to Anderson [38].

Notice that any permutation of $u_p$ does not change $x_\alpha$, *y*. Let us show that this transformation is a bijection a.s. between the quotient of ${\mathbb {C}}^n$ by the symmetric group $S_n$ and the space ${\mathbb {C}}^n$ of vectors $(x_1,\dots ,x_{n-1},y)$. For this purpose, we consider the following rational function:

$$\begin{aligned} Q(t):=\frac{\prod \limits _{1\le p\le n} (t-u_p)}{\prod _{1\le \beta \le n-1}(t-z_\beta )} \end{aligned}$$

If $z_\alpha$ are pairwise different, then

$$\begin{aligned} Q(t) =t-\sum \limits _{p=1}^{n} u_p+\sum \limits _{\alpha =1}^{n-1} z_\alpha + \sum \limits _{\alpha =1}^{n-1} \frac{\mathop {\textrm{res}}\nolimits \limits _{t=z_\alpha } Q(t)}{t-z_\alpha }. \end{aligned}$$

The residues are $\mathop {\textrm{res}}\nolimits _{t=z_\alpha }= -x_\alpha$. So

$$\begin{aligned} Q(t)=t-y-\sum _{1\le \alpha \le n-1} \frac{x_\alpha }{t-z_\alpha }, \end{aligned}$$

and *Q*(*t*) is uniquely determined by $x_\alpha$ and *y*. $\square$

Step 2.

#### Lemma 2

The complex Jacobian of the map (2.5)–(2.6) is

$$\begin{aligned} J(u;z)=\frac{\prod \limits _{1\le p\le q\le n}(u_p-u_q)}{\prod \limits _{1\le \alpha <\beta \le n-1}(z_\beta -z_\alpha )}. \end{aligned}$$

#### Proof

The statement is Lemma 2.3.c from [9]. It is a simple calculation, which is reduced to the Cauchy determinant, see, e.g., [36], Sec. I.4, Example 6.

So the real Jacobian of the transformation is $|J(u;z)|^2=J(u;z)^{{\varvec{1}}}$.

Denote by *I*(*u*; *z*) the integrand in (2.4). Then our integral is

2.7 $$\begin{aligned} n!\int _{{\mathbb {C}}^n} I\bigl (u(x,y);z\bigr ) J\bigl (u(x,y);z\bigr )^{-{{\varvec{1}}}}\,\,d\,{\overline{\overline{x}}} \,\,d\,{\overline{\overline{y}}} . \end{aligned}$$

The factor *n*! arises since the map $(u_1,\dots ,u_n)\mapsto (y,x_1,\dots ,x_{n-1})$ is an *n*!-sheeted (ramified) covering. We must express our integrand in the variables *x*, *y*.

Step 3. Let us show that for any $a\in {\mathbb {C}}$ we have

2.8 $$\begin{aligned} \prod _{p=1}^n (u_p+a)=\left( a+y-\sum _{\alpha =1}^{n-1} \frac{x_\alpha }{z_\alpha +a}\right) \prod _{\alpha =1}^{n-1} (z_\alpha +a). \end{aligned}$$

This is an identity ‘sum of residues is 0’ for the rational function

$$\begin{aligned} R(t)=\frac{\prod \limits _{1\le p\le n}(t-u_p)}{(t+a) \prod \limits _{1\le \beta \le n-1}(t-z_\beta )}. \end{aligned}$$

Indeed, the residues of *R*(*t*) are

$$\begin{aligned} \mathop {\textrm{res}}\nolimits \limits _{t=z_\alpha }R(t)&=-\frac{x_\alpha }{z_\alpha +a},\qquad \mathop {\textrm{res}}\nolimits \limits _{t=\infty } R(t)=y+a,\\ \mathop {\textrm{res}}\nolimits \limits _{x=-a}R(t)&= -\frac{\prod \limits _{1\le p\le n}(u_p+a)}{\prod \limits _{1\le \beta \le n-1}(z_\beta +a)}. \end{aligned}$$

Step 4. Now we are ready to transform the integrand in (2.7). Setting $a=0$, $-1$, $-z_\alpha$ in (2.8) we, respectively, get

$$\begin{aligned} \prod _p u_p^{{\varvec{\sigma }}-{{\varvec{1}}}}&=\prod _{\alpha =1}^{n-1} (z_\alpha )^{{\varvec{\sigma }}-{{\varvec{1}}}} \left( y-\sum _{\alpha =1}^{n-1} \frac{x_\alpha }{z_\alpha }\right) ^{{\varvec{\sigma }}-{{\varvec{1}}}};\\ \prod _p (1- u_p)^{{\varvec{\tau }}-{{\varvec{1}}}}&= (-1)^{n({\varvec{\tau }}-{{\varvec{1}}})}\prod _{\alpha =1}^{n-1} (z_\alpha -1)^{{\varvec{\tau }}-{{\varvec{1}}}} \left( -1+ y-\sum _{\alpha =1}^{n-1} \frac{x_\alpha }{z_\alpha -1}\right) ^{{\varvec{\tau }}-{{\varvec{1}}}}\\&=\prod _{\alpha =1}^{n-1} (1-z_\alpha )^{{\varvec{\tau }}-{{\varvec{1}}}} \left( 1- y-\sum _{\alpha =1}^{n-1} \frac{x_\alpha }{1-z_\alpha }\right) ^{{\varvec{\tau }}-{{\varvec{1}}}};\\ \prod _{p=1}^n (u_p-z_\alpha )^{{\varvec{\theta }}_\alpha -{{\varvec{1}}}}&= \left( -x_\alpha {\prod _{1\le \beta \le n-1, \beta \ne \alpha }(z_\beta -z_\alpha )}\right) ^{{\varvec{\theta }}_\alpha -{{\varvec{1}}}}. \end{aligned}$$

The factor $\prod (u_p-u_q)^{{\varvec{1}}}$ appears in the numerator of $I(\cdot )$ and the denominator of $J(\cdot )^{-{{\varvec{1}}}}$ and denominator, so it cancels. So we come to the expression

$$\begin{aligned} A\cdot \int _{{\mathbb {C}}^n} \left( y-\sum _{1\le \alpha \le n-1}\frac{x_\alpha }{z_\alpha }\right) ^{{\varvec{\sigma }}-{{\varvec{1}}}} \left( 1-y-\sum _{1\le \alpha \le n-1}\frac{x_\alpha }{1-z_\alpha }\right) ^{{\varvec{\tau }}-{{\varvec{1}}}} \prod _{\alpha =1}^{n-1} x_\alpha ^{{\varvec{\theta }}_\alpha -{{\varvec{1}}}}\,\,d\,{\overline{\overline{x}}} \, \,d\,{\overline{\overline{y}}} , \end{aligned}$$

where

$$\begin{aligned} A= & {} (-1)^{\sum \limits _{\alpha =1}^{n-1} {\varvec{\theta }}_\alpha }\, n! \\{} & {} \times \prod _{\alpha =1}^{n-1} z_\alpha ^{{\varvec{\sigma }}-{{\varvec{1}}}}(1-z_\alpha )^{{\varvec{\tau }}-{{\varvec{1}}}} \!\! \prod _{1\le \alpha ,\beta \le n-1,\,\alpha \ne \beta } (z_\beta -z_\alpha )^{{\varvec{\theta }}_\alpha -{{\varvec{1}}}}\!\! \prod _{1\le \alpha <\beta \le n-1} (z_\beta -z_\alpha )^{{{\varvec{1}}}} \end{aligned}$$

(the last two products are united in the final formula (2.4)).

Step 5. Changing the variables to

$$\begin{aligned} s=y-\sum _{\alpha =1}^{n-1} \frac{x_\alpha }{z_\alpha },\qquad t_\alpha =\frac{x_\alpha }{z_\alpha (1-z_\alpha )}, \end{aligned}$$

we come to the expression

$$\begin{aligned} A\cdot \prod _{\alpha =1}^{n-1}(z_\alpha (1-z_\alpha ))^{{\varvec{\theta }}_\alpha } \int _{{\mathbb {C}}^n} s^{{\varvec{\sigma }}-{{\varvec{1}}}} \prod _{\alpha =1}^{n-1} t_\alpha ^{{\varvec{\theta }}_\alpha -{{\varvec{1}}}}\cdot \left( 1-s-\sum _{\alpha =1}^{n-1} t_j\right) ^{{\varvec{\tau }}-{{\varvec{1}}}}\,\,d\,{\overline{\overline{t}}} \,\,d\,{\overline{\overline{s}}} . \end{aligned}$$

Applying the Dirichlet integral (2.2) we get the desired statement. $\square$

### Proof of Theorem 1

Denote

$$\begin{aligned}&\widehat{\varvec{\sigma }}_k:=\{{\varvec{\sigma }}_j\}_{j=1,\dots , k}, \qquad \widehat{\varvec{\tau }}_k=\{{\varvec{\tau }}_j\}_{j=1,\dots , k},\\&\widehat{\varvec{\theta }}_k=\{{\varvec{\theta }}_{j\alpha }\}_{j=1,\dots ,k-1;\,\alpha =1,\dots ,j}. \end{aligned}$$

Denote the integral (1.8) by $H(\widehat{\varvec{\sigma }}_n,\widehat{\varvec{\tau }}_n,\widehat{\varvec{\theta }}_n)$. We integrate in the variables $z_{n1}$, ..., $z_{nn}$ with Lemma 1 and come to

$$\begin{aligned} H(\widehat{\varvec{\sigma }}_n,\widehat{\varvec{\tau }}_n,\widehat{\varvec{\theta }}_n)= & {} H(\widehat{\varvec{\sigma }}_{n-1},\widehat{\varvec{\tau }}_{n-1},\widehat{\varvec{\theta }}_{n-1}) \\ {}{} & {} \times (-1)^{\sum \limits _{\alpha =1}^{n-1}{\varvec{\theta }}_{(n-1)\alpha }}\cdot \pi ^n n! \cdot \frac{\Gamma ^{\mathbb {C}}({\varvec{\sigma }}_n)\,\Gamma ^{\mathbb {C}}({\varvec{\tau }}_n)\prod \limits _{\alpha =1}^{n-1} \Gamma ^{\mathbb {C}}({\varvec{\theta }}_{(n-1)\alpha })}{\Gamma ^{\mathbb {C}}({\varvec{\sigma }}_n+{\varvec{\tau }}_n+\sum \limits _{\alpha =1} ^{n-1}{\varvec{\theta }}_{(n-1)\alpha })}. \end{aligned}$$

Repeating the same operation we come to the desired statement.

### A dual lemma

The following will be used to prove Lemma 5.

#### Lemma 3

2.9 $$\begin{aligned}{} & {} \int _{{\mathbb {C}}^n}\prod _{1\le \alpha \le n,\,1\le p\le n+1 } (z_\alpha -u_p)^{{\varvec{\theta }}-{{\varvec{1}}}}\prod _{1\le \alpha <\beta \le n} (z_\beta -z_\alpha )^{{\varvec{1}}}\,\,d\,{\overline{\overline{z}}} = \nonumber \\{} & {} \quad =n!\,\pi ^n\,\frac{\Gamma ^{\mathbb {C}}({\varvec{\theta }})^{n+1}}{\Gamma ^{\mathbb {C}}\bigl ((n+1){\varvec{\theta }}\bigr )}\cdot \prod _{1\le p,q\le n+1;\, p\ne q} (u_p-u_q)^{{\varvec{\theta }}-\frac{{{\varvec{1}}}}{{{\varvec{2}}}}}. \end{aligned}$$

The condition of convergence of this integral is

$$\begin{aligned} 0<\lfloor {\varvec{\theta }}\rfloor <\frac{1}{n+1}. \end{aligned}$$

#### Proof

Define new variables $w_1$, ..., $w_{n+1}$ by

$$\begin{aligned} w_p:=\frac{\prod \limits _{1\le \alpha \le n}(z_\alpha -u_p)}{\prod \limits _{1\le q\le n+1,q\ne p}(u_q-u_p)}, \end{aligned}$$

they are residues of the function

$$\begin{aligned} H(t)=\frac{\prod \limits _{1\le \alpha \le n}(t-z_\alpha )}{\prod \limits _{1\le p\le n+1}(t-u_p)} \end{aligned}$$

at points $t=u_p$. The residue at $\infty$ is $-1$, and therefore $\sum w_p=1$. On the other hand for fixed $u_p$ a function *H*(*t*) is uniquely determined by its residues, and therefore $w_p$ uniquely determine $z_\alpha$ up to a permutation.

We choose $w_1$, ..., $w_n$ as independent variables. $\square$

#### Lemma 4

The complex Jacobian of the transformation $(z,\dots ,z_n)\mapsto (w_1,\dots , w_n)$ is

$$\begin{aligned} J(z;u)= \det \limits _{1\le p,\,\alpha \le n}\Bigl \{\frac{\partial w_p}{\partial z_\alpha }\Bigr \} = \frac{\prod \limits _{1\le \alpha<\beta \le n}(z_\beta -z_\alpha )}{\prod \limits _{1\le p<q\le n+1}(u_p-u_q)}. \end{aligned}$$

#### Proof of Lemma 4

We have

$$\begin{aligned} \frac{\partial w_p}{\partial z_\alpha }= \frac{w_p}{z_\alpha -u_p}, \end{aligned}$$

So,

$$\begin{aligned} J(z;u)= & {} \prod _{1\le p\le n} w_p\cdot \det \limits _{1\le \alpha ,\,p\le n} \Bigl \{\frac{1}{z_\alpha -u_p}\Bigr \}\\= & {} \frac{\prod \limits _{1\le \alpha ,p\le n}(z_\alpha -u_p)}{\prod \limits _{1\le p, q\le n+1,q\ne p}(u_q-u_p)} \cdot \frac{\prod \limits _{1\le \alpha<\beta \le n}(z_\alpha -z_\beta ) \prod \limits _{1\le p<q\le n}(u_p-u_q)}{\prod \limits _{1\le \alpha ,p\le n}(z_\alpha -u_p)}. \end{aligned}$$

Here we applied the formula for the Cauchy determinant $\det \{\frac{1}{z_\alpha -u_p}\}$ (see, e.g., [36], Sect I.4, Example 6). After a cancelation we come to the desired formula.

The real Jacobian is $J(z;w)^{{{\varvec{1}}}}$.

Next, we change variables $(z_1,\dots ,z_n)\mapsto (w_1,\dots , w_n)$ in (2.9). We have

$$\begin{aligned} (z_\beta -z_\alpha )^{{\varvec{1}}} \cdot J(z;u)^{-{{\varvec{1}}}}&=\prod _{1\le p<q\le n}(u_p-u_q)^{{\varvec{1}}};\\ \prod _{1\le \alpha \le n,1\le p\le n+1 } (z_\alpha -u_p)^{{\varvec{\theta }}-{{\varvec{1}}}}&=\prod _{p=1}^{n+1} w_p^{{\varvec{\theta }}-{{\varvec{1}}}}\cdot \prod _{1\le p<q\le n}(u_p-u_q)^{2{\varvec{\theta }}-{{\varvec{2}}}}. \end{aligned}$$

Now the integral in the left hand side of (2.9) converts to

$$\begin{aligned} n! \prod _{1\le p< q\le n+1}(u_p-u_q)^{2{\varvec{\theta }}-{{\varvec{1}}}} \int _{{\mathbb {C}}^n} \prod _{p=1}^{n+1} w_p^{{\varvec{\theta }}-{{\varvec{1}}}} \,\,d\,{\overline{\overline{w_1}}} \dots \,d\,{\overline{\overline{w_n}}} . \end{aligned}$$

We remember that $w_{n+1}=1-\sum \limits _{p\le n} w_p$ and come to the Dirichlet integral (2.2). This completes the proof of Lemma 3. $\square$

### Proof of Theorem 2

Fix $m\le n$. Let $k< m$. Denote $\,d\,{\overline{\overline{\mathcal {Z}}}} :=\prod _{j=k}^{n} \prod _{\alpha =1}^j \,d\,{\overline{\overline{z}}} _{j\alpha }$. Denote

2.10 $$\begin{aligned} H_n^{m,k}:= & {} \int \limits _{\mathcal {C}_{\,n}^{\,\,k}} \prod _{j=k}^{m-1} \frac{\prod \limits _{1\le \alpha \le j;\, 1\le p\le j+1}(z_{j\alpha }-z_{(j+1)p})^{{\varvec{\nu }}-{{\varvec{1}}} }}{\prod \limits _{1\le \alpha ,\beta \le j,\,\alpha \ne \beta }(z_{j\alpha }-z_{j\beta })^{{\varvec{\nu }}-{{\varvec{1}}}}}\nonumber \\{} & {} \times \prod _{1\le \alpha ,\beta \le k,\, \alpha \ne \beta } (z_{k\alpha }-z_{k\beta })^{{\varvec{\nu }}-\frac{{{\varvec{1}}}}{{{\varvec{2}}}}} \nonumber \\{} & {} \times \prod _{j=m}^{n-1}\prod _{\alpha =1}^j z_{j\alpha }^{{\varvec{\sigma }}_j-{\varvec{\sigma }}_{j+1}-{\varvec{\theta }}_{j\alpha }}(1-z_{j\alpha }) ^{{\varvec{\tau }}_j-{\varvec{\tau }}_{j+1}-{\varvec{\theta }}_{j\alpha }} \prod _{p=1}^n z_{np}^{{\varvec{\sigma }}_n-{{\varvec{1}}}}(1-z_{np})^{{\varvec{\tau }}_n-{{\varvec{1}}}} \nonumber \\{} & {} \times \prod _{j=m}^{n-1} \frac{\prod \limits _{1\le \alpha \le j;\, 1\le p\le j+1}(z_{j\alpha }-z_{(j+1)p})^{{\varvec{\theta }}_{j\alpha }-{{\varvec{1}}}}}{\prod \limits _{1\le \alpha ,\beta \le j;\,\alpha \ne \beta }(z_{j\alpha }-z_{j\beta })^{{\varvec{\theta }}_{j\alpha }-{{\varvec{1}}}}}\nonumber \\{} & {} \times \prod _{1\le p<q\le n}(z_{np}-z_{nq})^{{{\varvec{1}}}}\,\, \,d\,{\overline{\overline{\mathcal {Z}}}} . \end{aligned}$$

Since we have a cancelation in the first and second lines of the integrand, we can replace these rows by

2.11 $$\begin{aligned} \prod \limits _{1\le \alpha \le k;\, 1\le p\le k+1}(z_{k\alpha }-z_{(k+1)p})^{{\varvec{\nu }}-{{\varvec{1}}}} \cdot \prod \limits _{1\le \alpha <\beta \le k} (z_{k\alpha }-z_{k\beta })^{{{\varvec{1}}} } \times \nonumber \\ \times \prod _{j=k+1}^{m-1} \frac{\prod \limits _{1\le \alpha \le j;\, 1\le p\le j+1}(z_{j\alpha }-z_{(j+1)p})^{{\varvec{\nu }}-{{\varvec{1}}} }}{\prod \limits _{1\le \alpha ,\beta \le j,\,\alpha \ne \beta }(z_{j\alpha }-z_{j\beta })^{{\varvec{\nu }}-{{\varvec{1}}}}}. \end{aligned}$$

If $k=1$, then we have an integral over $\mathcal {C}_n$ of the form (1.8) with parameters

$$\begin{aligned}&({\varvec{\sigma }}_m+(m-1){\varvec{\nu }},{\varvec{\sigma }}_m+(m-2){\varvec{\nu }}, \dots ,{\varvec{\sigma }}_m+2{\varvec{\nu }},{\varvec{\sigma }}_m+{\varvec{\nu }}, {\varvec{\sigma }}_m, {\varvec{\sigma }}_{m+1},{\varvec{\sigma }}_{m+2}, \dots , {\varvec{\sigma }}_n),\\&({\varvec{\tau }}_m+(m-1){\varvec{\nu }},{\varvec{\tau }}_m+(m-2){\varvec{\nu }}, \dots ,{\varvec{\tau }}_m+2{\varvec{\nu }},{\varvec{\tau }}_m+{\varvec{\nu }}, {\varvec{\tau }}_m, {\varvec{\tau }}_{m+1},{\varvec{\tau }}_{m+2}, \dots , {\varvec{\tau }}_n),\\&\bigl (\{{\varvec{\nu }}\}, \{{\varvec{\nu }},{\varvec{\nu }}\},\dots ,\underbrace{\{{\varvec{\nu }},\dots {\varvec{\nu }}\}} _{m-1 \text {times}},\\&\qquad \qquad \qquad \{{\varvec{\theta }}_{m\alpha }\}_{\alpha =1,\dots ,m}, \{{\varvec{\theta }}_{(m+1)\alpha }\}_{\alpha =1,\dots ,m+1},\dots , \{{\varvec{\theta }}_{(n-1)\alpha }\}_{\alpha =1,\dots ,n-1}\bigr ). \end{aligned}$$

Therefore, by Theorem 1

2.12 $$\begin{aligned} H_n^{m,1}= & {} (-1)^{m(m-1){\varvec{\nu }}/2+\sum \limits _{j=m}^{n-1}\sum \limits _{\alpha =1}^j {\varvec{\theta }}_{j\alpha }}\, \pi ^{n(n+1)/2}\, \prod \limits _{j=1}^n j! \nonumber \\{} & {} \times \prod \limits _{j=1}^{m-1} \frac{\Gamma ^{\mathbb {C}}({\varvec{\sigma }}_m+(m-j){\varvec{\nu }})\, \Gamma ^{\mathbb {C}}({\varvec{\tau }}_m+(m-j){\varvec{\nu }})}{\Gamma ^{\mathbb {C}}({\varvec{\sigma }}_m+{\varvec{\tau }}_m+(2m-j-1){\varvec{\nu }})} \cdot \Gamma ^{\mathbb {C}}({\varvec{\nu }})^{m(m-1)/2}\nonumber \\{} & {} \times \prod \limits _{j=m}^{n-1}\prod _{\alpha =1}^j \Gamma ^{\mathbb {C}}({\varvec{\theta }}_{j\alpha }) \prod _{j=m}^n\frac{\Gamma ^{\mathbb {C}}({\varvec{\sigma }}_j)\,\Gamma ^{\mathbb {C}}({\varvec{\tau }}_j)}{\Gamma ^ {\mathbb {C}}({\varvec{\sigma }}_j+{\varvec{\tau }}_j+\sum \limits _{\alpha =1}^{j-1}{\varvec{\theta }}_{(j-1)\alpha })}. \end{aligned}$$

#### Lemma 5

$$\begin{aligned} H_n^{m,k}&=H_n^{m,k+1}\cdot \pi ^k k! \frac{\Gamma ^{\mathbb {C}}({\varvec{\nu }})^{k+1}}{\Gamma ^{\mathbb {C}}((k+1){\varvec{\nu }})},\qquad \text {for} k<m-1;\\ H_n^{m,m-1}&=J_n^m(\widehat{\varvec{\sigma }}_n^m,\widehat{\varvec{\tau }}_n^m;{\varvec{\nu }}, \widehat{\varvec{\theta }}_n^m)\cdot \pi ^{m-1} (m-1)!\,\, \frac{\Gamma ^{\mathbb {C}}({\varvec{\nu }})^{m}}{\Gamma ^{\mathbb {C}}(m{\varvec{\nu }})}. \end{aligned}$$

#### Proof of Lemma 5

Denote $\{z_{m\alpha }\}:=(z_{m1}, \dots , z_{mm})$. Fix *k* to be $k\le m-1$. The integrand in (2.10) has the form

$$\begin{aligned} A\bigl (\{z_{k\alpha }\}, \{z_{(k+1)\alpha }\}\bigr )\, B\bigl (\{z_{(k+1)\alpha }\}, \{z_{(k+2)\alpha }\}, \dots , \{z_{n\alpha \}}\bigr ), \end{aligned}$$

where $A(\dots )$ is the first line of (2.11) and $B(\dots )$ does not depend on variables $z_{k1}, \dots , z_{kk}$ (it is the product of the second line of (2.11) and the third and fourth lines of (2.10)). So integration with respect to the variables $z_{k\alpha }$ gives

$$\begin{aligned} \int _{{\mathbb {C}}^k} A\bigl (\{z_{k\alpha }\}, \{z_{(k+1)\alpha }\}\bigr )\, \,d\,{\overline{\overline{z}}} _{k1} \dots \,d\,{\overline{\overline{z}}} _{kk} \times B\bigl (\{z_{(k+1)\alpha }\}, \dots , \{z_{n\alpha }\}\bigr ). \end{aligned}$$

We apply Lemma 3 and come to

$$\begin{aligned} \pi ^k k! \frac{\Gamma ^{\mathbb {C}}({\varvec{\nu }})^{k+1}}{\Gamma ^{\mathbb {C}}((k+1){\varvec{\nu }})} \cdot \Bigl [ \prod _{\begin{array}{c} 1\le \alpha ,\beta \le k+1\\ \alpha \ne \beta \end{array}} (z_{(k+1)\alpha }-z_{(k+1)\beta })^{{\varvec{\nu }}-\frac{{{\varvec{1}}}}{{{\varvec{2}}}}} \cdot B\bigl (\{z_{(k+1)\alpha }\}, \dots , \{z_{n\alpha }\}\bigr ) \Bigr ]. \end{aligned}$$

The expression in the big square brackets is the integrand of $H_n^{m,k+1}$ in formula (2.10), when $k<m-1$ and the integrand in $J_n^m(\widehat{\varvec{\sigma }}_n^m,\widehat{\varvec{\tau }}_n^m;{\varvec{\nu }},\widehat{\varvec{\theta }}_n^m)$ in formula (1.9) when $k=m-1$. $\square$

Combining Lemma 5 with expression (2.12) for $H_{n}^{m,1}$, we come to formula (1.10). The conditions of convergence of the $H_{n}^{m,1}$ are

$$\begin{aligned}&\lfloor {\varvec{\theta }}_{j\alpha }\rfloor>0, \,\, \left\lfloor {\varvec{\tau }}_j\right\rfloor>0,\,\, \lfloor {\varvec{\sigma }}_j\rfloor>0, \,\, \lfloor {\varvec{\sigma }}_j+{\varvec{\tau }}_j+\sum _{\alpha =1}^{j-1}{\varvec{\theta }}_{(j-1)\alpha } \rfloor<1, \qquad \text {for} j> m;\\&\lfloor {\varvec{\nu }}\rfloor >0, \,\, \lfloor m{\varvec{\nu }}\rfloor<1,\,\, \lfloor {\varvec{\sigma }}_m+{\varvec{\tau }}_m+2(m-1){\varvec{\nu }}\rfloor <1, \end{aligned}$$

and these conditions are sufficient for convergence of (1.9).

### The Dotsenko–Fateev–Aomoto integral

Let $m=n$. Then in (1.9) we have integration $\,d\,{\overline{\overline{z}}} _{n1}, \dots , \,d\,{\overline{\overline{z}}} _{nn}$, two factors $\prod _{j=m}^{n-1}$ disappear. The integrand transforms to

$$\begin{aligned} \prod _{p=1}^n z_{np}^{{\varvec{\sigma }}_n-{{\varvec{1}}}}(1-z_{np})^{{\varvec{\tau }}_n-{{\varvec{1}}}} \cdot \prod _{1\le \alpha ,\beta \le n;\, \alpha \ne \beta } (z_{n\beta }-z_{n\alpha })^{{\varvec{\nu }}-\frac{{{\varvec{1}}}}{{{\varvec{2}}}}} \cdot \prod _{1\le p<q\le n} (z_{np}-z_{nq})^{{\varvec{1}}}. \end{aligned}$$

We unite the second and third factors and come to the desired form (1.13).

## Data Availability

There are no extra data and materials related to the paper.

## Footnotes

1 On beta integrals, see Askey [10], Andrews, Askey, Roy [1], on matrix beta integrals, see a review [11]. Beta integrals are integrals whose integrands are products (for instance, of power functions or Gamma functions) and the right-hand side is a product of Gamma functions (or *q*-Gamma function). Quite often such integrands are weight functions for orthogonal polynomials or are related to explicitly solvable spectral problems with continuous or partially continuous spectra.

2 There are two groups of authors who worked on complex twins of hypergeometric functions and beta integral, mathematicians motivated by representations of the Lorentz group and mathematical physicists, who also partially are motivated by representations of the Lorentz group.

3 These spaces are spaces of positive definite matrices of size *n* over ${\mathbb {R}}$, ${\mathbb {C}}$, ${\mathbb {H}}$.

4 See Sekiguchi [35], Macdonald [36], Sect. VII.3, Example 3.

5 These spaces are complexifications of the spaces (1.16). They are spaces of non-degenerate complex matrices, precisely, symmetric matrices of size *n*, square matrices of size *n*, skew-symmetric matrices of size 2*n*. On spherical distributions on symmetric spaces, see Heckman, Schlichtkrull [37], Chapters 2, 3.
